# Association between *VEGF* gene polymorphisms (11 sites) and polycystic ovary syndrome risk

**DOI:** 10.1042/BSR20191691

**Published:** 2020-03-13

**Authors:** Li Huang, Lunwen Wang

**Affiliations:** 1 Department of Obstetrics and Gynecology, Xishui Hospital, Affiliated to Hubei Institute of Science and Technology, China; 2 Department of Respiratory Medicine, Xishui Hospital, Affiliated to Hubei Institute of Science and Technology, China

**Keywords:** meta-analysis, polycystic ovary syndrome, polymorphism, VEGF

## Abstract

Vascular endothelial growth factor (*VEGF*) plays a critical role in ovarian folliculogenesis and normal reproductive function. So far, several studies focusing on association between *VEGF* gene polymorphisms and polycystic ovary syndrome (PCOS). However, above association between the *VEGF* gene polymorphisms and PCOS susceptibility is uncertain. Hence, we performed a timely meta-analysis containing all current publications to make clear this relationship. We searched articles from the PubMed, Embase and Chinese language (WanFang and CNKI) databases that were published up until May 10, 2019. Finally, we obtained 9 studies, containing 29 case–control studies and 11 different polymorphisms. The odds ratios (OR) and 95% confidence intervals (CI) were revealed association strengths. There were significantly decreased associations between rs2010963 (-634), +9812, +405 polymorphisms and PCOS risk. Nevertheless, there existed increased associations between rs699947 (-2578), rs833061, rs1570360 (-1154), rs3025020, rs3025039 polymorphisms and PCOS susceptibility. Our current analysis suggested *VEGF* gene polymorphisms may be associated with PCOS risk, which is possible to be expected to become biomarkers of early detection for women.

## Introduction

Polycystic ovarian syndrome (PCOS) is a highly prevalent disorder affecting multiple aspects of a women’s overall health, with long-term effects that transcend well beyond the reproductive age [1–3]. Clinically, PCOS is characterized by hyperandrogenism manifested by hirsutism, acne and androgenic alopecia [4]. Patients with PCOS demonstrate reproductive abnormalities [5], marked insulin resistance [6], increased risk for Type 2 diabetes mellitus [7], coronary heart disease [8], atherogenic dyslipidemia [9], cerebrovascular morbidity [10], and anxiety and depression [11]. Although it was first reported in 1935 by Stein et al., the etiology remains unclear. Data from many studies suggest that genetics is very important in the development of PCOS [12].

Vascular endothelial growth factor (VEGF) gene, also known as VEGFA, VPF and MVCD1, locates at 6p21.1 and contains nine exon counts, and is a member of the PDGF/VEGF growth factor family [13]. It encodes a heparin-binding protein, which exists as a disulfide-linked homodimer. This growth factor induces proliferation and migration of vascular endothelial cells and is essential for both physiological and pathological angiogenesis [14].

VEGF plays a critical role in ovarian folliculogenesis and normal reproductive function [14], highlighted by the findings that women with PCOS had increased serum levels of VEGF, which paralleled increases in Doppler flow velocities within ovarian vessels [15,16]. High vascularization may result in abnormal growth of the theca interna, the site of androgen steroidogenesis, leading to hyperandrogenism, a hallmark of PCOS [17]. Several single-nucleotide polymorphisms (SNPs) were identified within the VEGF gene, of which some were functional, directly affecting VEGF secretion and its serum expression [18–20].

So far, many studies have investigated the association between *VEGF* polymorphisms and PCOS risk. However, the results were not conclusive or consistent. Considering the vital role of *VEGF* gene in the development of PCOS, we conducted a timely meta-analysis including 11 SNPs [21–29] to derive a more comprehensive estimation of the association between *VEGF* gene polymorphisms and PCOS susceptibility to identify some significant biomarkers.

## Materials and methods

### Identification and eligibility of relevant studies

We applied the PubMed, Embase, WanFang and CNKI databases using the key words ‘VEGF or VEGFA or VPF’, ‘PCOS or Polycystic ovarian syndrome’ and ‘polymorphism’ or ‘variant’ to identify including studies. The last search was updated on May 10, 2019. Finally, 29 case–control studies about 11 different SNPs were retrieved.

### Inclusion criteria and exclusion criteria

Including studies had to meet following criteria: (1) address the correlation between PCOS risk and the *VEGF* gene SNPs; (2) be a case–control study, and (3) have sufficient genotype (wild-type and mutant type) numbers in each case and control group. The following exclusion criteria were used: (1) lack of a control population; (2) lack of available genotype frequency data; and (3) duplicated studies.

### Data extraction

The following items were selected: the first author’s last name, the year of publication, the country of origin, the ethnicity of subjects, SNP type, total number of case and control groups, source of control (SOC), the number of each genotype frequency in the case/control groups, the Hardy–Weinberg equilibrium (HWE) in the control group, and the genotyping method. Ethnicity was categorized as Asian, European, Mixed and African.

### Statistical analysis

Odds ratio (OR) with 95% confidence intervals (CI) were used to measure the strength of the association between the *VEGF* gene SNPs and PCOS risk. The statistical significance of the summary OR was determined with the *Z*-test. A heterogeneity assumption was evaluated among studies using a chi-square-based *Q*-test. If a *P-*value of < 0.10 for the *Q*-test indicated heterogeneity among the studies. If significant heterogeneity was detected, the random-effects model (DerSimonian-Laird method) was used. Otherwise, the fixed-effects model (Mantel–Haenszel method) was applied [30,31].

We investigated the relationship between genetic variants of the *VEGF* gene SNPs and PCOS risk by the allelic contrast (1 vs. 2), homozygote comparison (1/1 vs. 2/2), dominant genetic model (1/1+1/2 vs. 2/2), heterozygote comparison (1/2 vs. 2/2) and recessive genetic model (1/1 vs. 1/2+2/2). A sensitivity analysis was performed by omitting studies, one after another, to assess the stability of results. The departure of the *VEGF* gene SNPs from expected frequencies under HWE was assessed in controls using the Pearson chi-square test (*P* < 0.05 was considered significant). Funnel plot asymmetry was assessed using Begg’s test and publication bias was assessed using Egger’s test [32], both the *P*-value < 0.05 is considered as significant. All statistical tests for this meta-analysis were performed with Stata software (version 11.0; StataCorp LP, College Station, TX).

### Network of gene interaction of *VEGF* gene

To more complete understanding of the role of VEGF in PCOS, the network of gene–gene interactions for VEGF gene was utilized through String online server (http://string-db.org/) [33].

## Results

### Study characteristics

**Figure 1:**
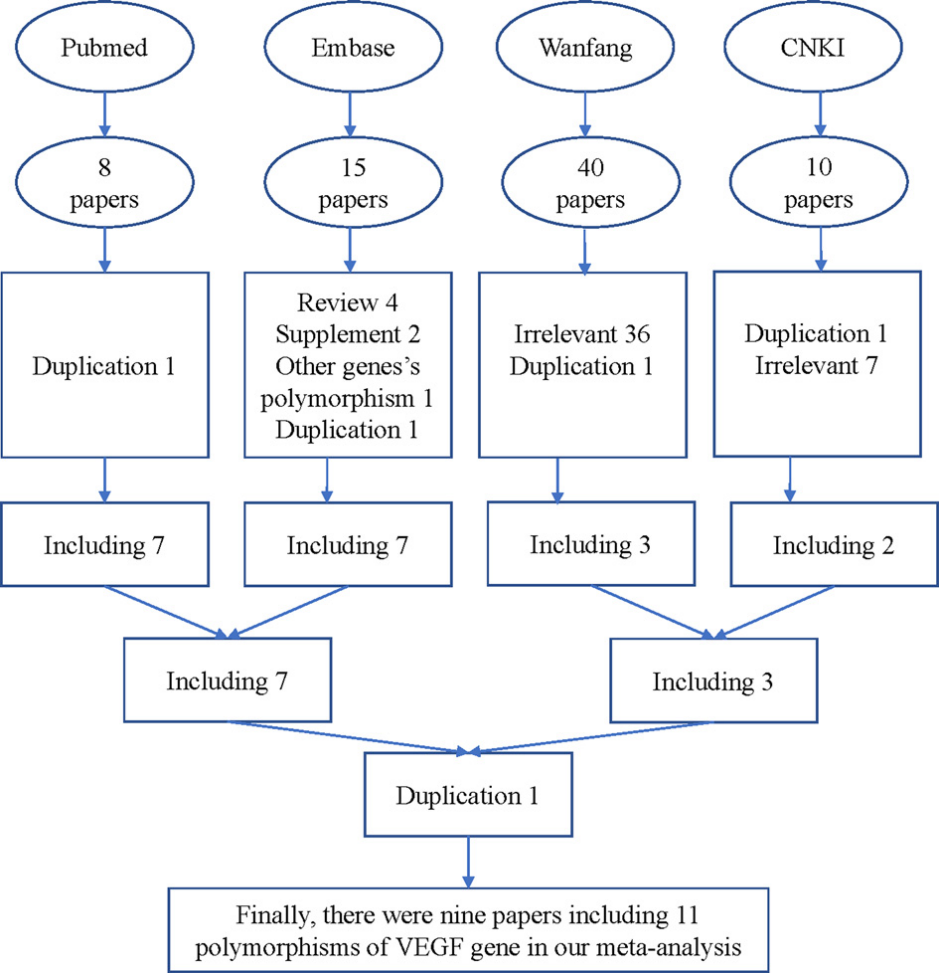
Flowchart illustrating the search strategy used to identify association studies for *VEGF* gene polymorphisms and PCOS risk

In total, 73 articles were collected from the PubMed, Embase, CNKI and WanFang databases via a literature search using different combinations of above keywords. As shown in Figure 1, 64 articles were excluded (such as duplications, irrelevant articles, reviews and other gene’s polymorphisms). Finally, 9 different articles including 11 SNPs were included in our current meta-analysis (Figure 1). Study characteristics from the published studies on the relationship between the *VEGF* gene SNPs and PCOS risk are summarized in Table 1. In all the studies, the controls were women under normal pregnancy. The detail of 11 SNPs were rs2010963 or -634 (three case–control studies including 632 cases and 622 controls), +9812 (two case–control studies including 212 cases and 183 controls), +13553 (two case–control studies including 208 cases and 184 controls), -460 (two case–control studies including 263 cases and 285 controls), +405 (two case–control studies including 263 cases and 285 controls), rs699947 or -2578 (four case–control studies including 724 cases and 782 controls), rs833061 (three case–control studies including 618 cases and 673 controls), rs1570360 or -1154 (four case–control studies including 697 cases and 737 controls), rs833068 (two case–control studies including 500 cases and 540 controls), rs3025020 (two case–control studies including 500 cases and 540 controls), and rs3025039 or +936 (three case–control studies including 586 cases and 628 controls).

**Table 1:** Basic information for included studies of the association between polymorphisms in *VEGF* gene and PCOS susceptibility

Author	Year	Country	Ethnicity	SNPs	Case	Control	SOC	Cases			Controls			HWE	
								1/1*	1/2*	2/2*	1/1*	1/2*	2/2*		Genotype
Lee	2008	Korea	Asian	rs2010963 (-634)	132	99	HB	26	60	46	20	45	34	0.47	TaqMan
Almawi	2016	Bahrain	Asian	rs2010963 (-634)	382	393	PB	57	142	183	42	190	161	0.01	TaqMan
Huang	2018	China	Asian	rs2010963 (-634)	118	130	HB	13	45	60	19	64	47	0.71	PCR-LDR
Lee	2008	Korea	Asian	+9812	132	99	HB	6	36	90	12	29	58	0.01	TaqMan
Ding	2009	China	Asian	+9812	80	84	HB	6	24	50	16	24	44	0.001	sequencing
Lee	2008	Korea	Asian	+13553	128	100	HB	4	35	89	10	31	59	0.06	TaqMan
Ding	2009	China	Asian	+13553	80	84	HB	0	35	45	0	30	54	0.046	sequencing
Vural	2009	Turkey	European	-460	137	155	HB	18	64	55	29	74	52	0.76	F-LHPLC
Guruvaiah	2014	India	Asian	-460	126	130	HB	27	59	40	25	72	33	0.2	sequencing
Vural	2009	Turkey	European	+405	137	155	HB	3	44	90	4	39	112	0.78	F-LHPLC
Guruvaiah	2014	India	Asian	+405	126	130	HB	10	46	70	19	59	52	0.73	sequencing
Salem	2016	Tunisia	African	rs699947(-2578)	118	150	HB	20	63	35	29	76	45	0.76	TaqMan
Almawi	2016	Bahrain	Asian	rs699947(-2578)	382	393	PB	64	183	135	50	178	165	0.85	TaqMan
Gomes	2019	Brazil	Mixed	rs699947(-2578)	87	84	HB	27	38	22	18	41	25	<0.001	PCR-RFLP
Vural	2009	Turkey	European	rs699947 (-2578)	137	155	HB	22	63	52	25	78	52	<0.001	F-LHPLC
Salem	2016	Tunisia	African	rs833061	118	150	HB	30	55	33	32	76	42	0.82	TaqMan
Almawi	2016	Bahrain	Asian	rs833061	382	393	PB	78	174	130	71	190	132	0.85	TaqMan
Huang	2018	China	Asian	rs833061	118	130	HB	10	45	63	8	42	80	0.44	PCR-LDR
Salem	2016	Tunisia	African	rs1570360 (-1154)	118	150	HB	19	42	57	18	57	75	0.17	TaqMan
Almawi	2016	Bahrain	Asian	rs1570360 (-1154)	382	393	PB	45	140	197	44	131	218	<0.001	TaqMan
Li	2014	China	Asian	rs1570360 (-1154)	110	110	HB	3	29	78	5	30	65	0.53	PCR-RFLP
Gomes	2019	Brazil	Mixed	rs1570360 (-1154)	87	84	HB	7	24	56	1	31	52	<0.001	TaqMan
Salem	2016	Tunisia	African	rs833068	118	150	HB	13	63	42	23	63	64	0.26	TaqMan
Almawi	2016	Bahrain	Asian	rs833068	382	390	PB	51	175	156	34	200	156	0.006	TaqMan
Salem	2016	Tunisia	African	rs3025020 (-583)	118	150	HB	10	40	68	8	52	90	0.89	TaqMan
Almawi	2016	Bahrain	Asian	rs3025020 (-583)	382	393	PB	54	149	179	35	155	203	0.49	TaqMan
Salem	2016	Tunisia	African	rs3025039 (+936)	118	150	HB	3	27	88	4	19	127	0.005	TaqMan
Almawi	2016	Bahrain	Asian	rs3025039 (+936)	382	393	PB	5	81	296	7	68	318	0.141	TaqMan
Gomes	2019	Brazil	Mixed	rs3025039 (+936)	86	85	HB	70	16		25	60			PCR-RFLP

HWE: Hardy–Weinberg equilibrium; HB: hospital-based; SOC: source of control; SNPs: single-nucleotide polymorphism; PCR-RFLP: polymerase chain reaction and restrictive fragment length polymorphism; PCR-LDR: polymerase chain reaction-ligase detection reaction; F-LHPLC: fluorescence-labeled hybridization probes in a Light-Cycler; * 1/1: mutant genotype, 1/2: heterozygous, 2/2: wide type.

### Quantitative synthesis

**Figure 2:**
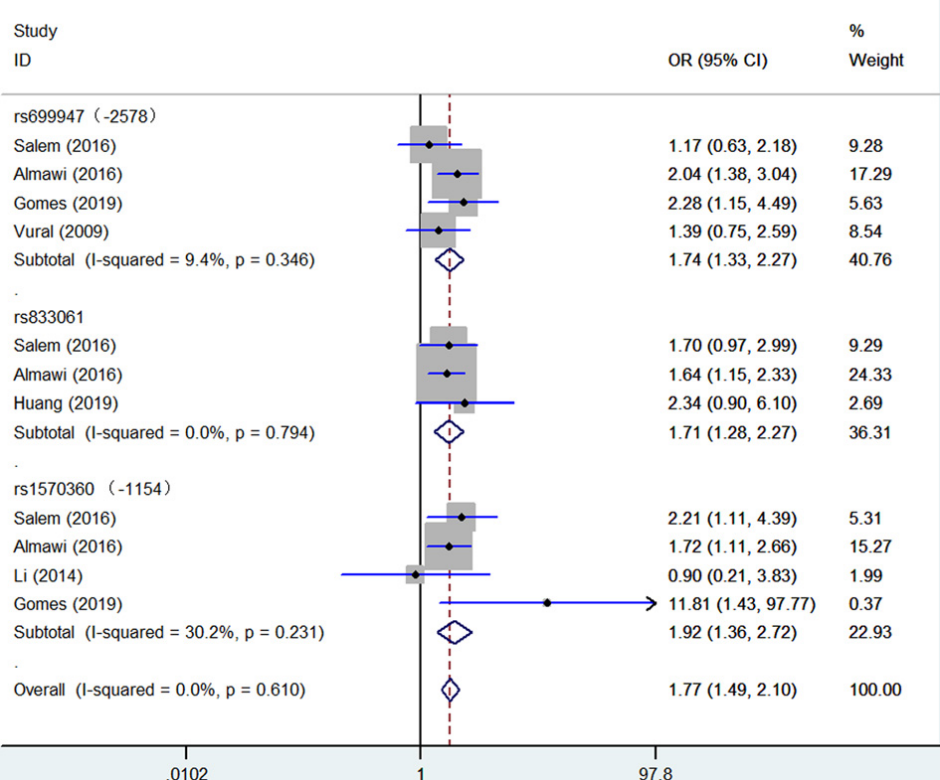
Forest plot of PCOS risk associated with *VEGF* gene polymorphisms (rs699947, rs833061, rs1570360) (Recessive model) in the whole The squares and horizontal lines correspond to the study-specific OR and 95% CI. The area of the squares reflects the weight (inverse of the variance). The diamond represents the summary OR and 95% CI.

**Figure 3:**
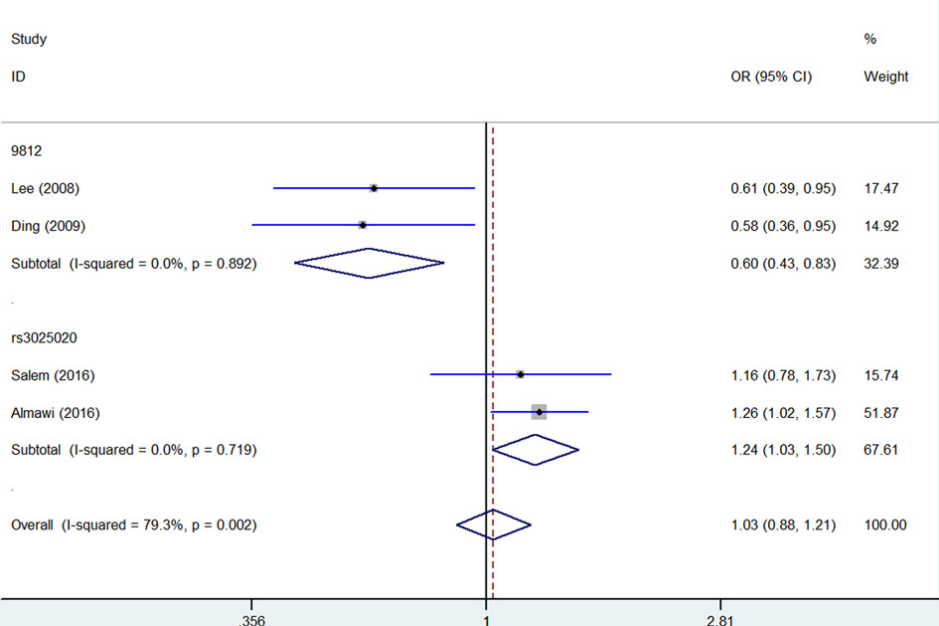
Forest plot of PCOS risk associated with *VEGF* gene polymorphisms (rs3025020 and +9812) (Homozygote comparison) in the whole The squares and horizontal lines correspond to the study-specific OR and 95% CI. The area of the squares reflects the weight (inverse of the variance). The diamond represents the summary OR and 95% CI.

**Figure 4:**
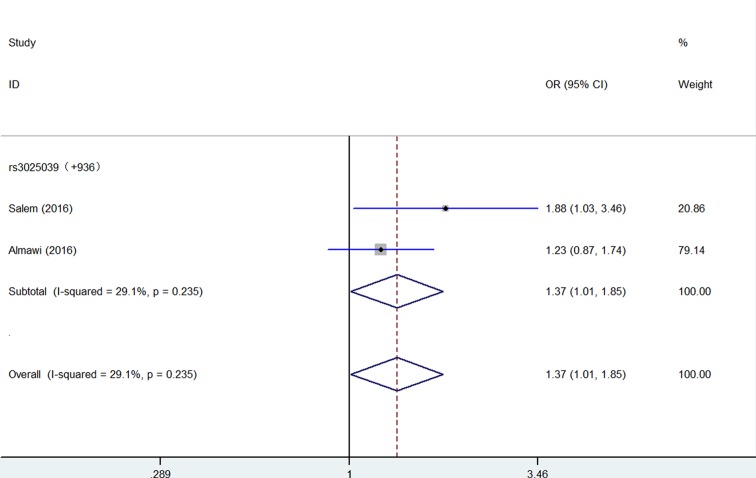
Forest plot of PCOS risk associated with *VEGF* gene polymorphism (rs3025039) (Dominant model) in the whole. The squares and horizontal lines correspond to the study-specific OR and 95% CI The area of the squares reflects the weight (inverse of the variance). The diamond represents the summary OR and 95% CI.

Significantly increased association were detected between five *VEGF* gene SNPs and PCOS susceptibility: rs699947 (Recessive model: OR = 1.74, 95% CI = 1.33–2.27, *P* = 0.346 for heterogeneity, *P* < 0.001, Figure 2, Table 2); rs833061 (Recessive model: OR = 1.71, 95% CI = 1.28–2.21, *P* = 0.794 for heterogeneity, *P* < 0.001, Figure 2, Table 2); rs1570360 (Recessive model: OR = 1.92, 95% CI = 1.36–2.72, *P* = 0.231 for heterogeneity, *P* < 0.001, Figure 2, Table 2); rs3025020 (Homo*z*ygote comparison: OR = 1.73, 95% CI = 1.13–2.65, *P* = 0.920 for heterogeneity, *P* = 0.011, Figure 3, Table 2); rs3025039 (Dominant model: OR = 1.37, 95% CI = 1.01–1.85, *P* = 0.235 for heterogeneity, *P* = 0.042, Figure 4, Table 2).

**Table 2:** Total and stratified subgroup analysis for *VEGF gene* polymorphisms and PCOS susceptibility

Variables	*N*	Case/	Allelic contrast			Homo*z*ygote comparison			Heterozygote comparison			Dominant model			Recessive model		
		Control	OR(95%CI)	*P*_h_	*P*	OR(95%CI)	*P*_h_	*P*	OR(95%CI)	*P*_h_	*P*	OR(95%CI)	*P*_h_	*P*	OR(95%CI)	*P*_h_	*P*
rs2010963 (-634)	3	632/622	0.89(0.75–1.05)	0.242	0.152	0.98(0.69–1.38)	0.233	0.899	**0.68(0.53–0.86)**	**0.339**	**0.002**	**0.74(0.59–0.93)**	**0.307**	**0.009**	**1.68(1.22–2.30)**	**0.197**	**0.001**
+9812	2	212/183	**0.60(0.43–0.83)**	**0.892**	**0.002**	**0.33(0.16–0.68)**	**0.974**	**0.003**	0.83(0.53–1.31)	0.838	0.425	0.66(0.44–0.99)	1.000	0.047	0.57(0.28–1.16)	0.985	0.119
+13553	2	208/184	0.86(0.40–1.85)	0.031	0.697			–	1.00(0.65–1.51)	0.153	0.991	0.93(0.42–2.02)	0.061	0.846			–
-460	2	263/285	0.84(0.66–1.07)	0.487	0.158	0.72(0.44–1.18)	0.413	0.194	0.75(0.51–1.10)	0.626	0.142	0.74(0.52–1.06)	0.938	0.105	1.19(0.77–1.84)	0.278	0.422
+405	2	263/285	0.86(0.41–1.77)	0.012	0.673	**0.48(0.23–1.00)**	**0.327**	**0.050**	0.90(0.38–2.15)	0.018	0.819	0.86(0.34–2.13)	0.009	0.731	0.85(0.42–1.73)	0.430	0.661
rs699947 (-2578)	4	724/782	1.13(0.98–1.31)	0.258	0.100	1.28(0.94–1.73)	0.333	0.112	1.17(0.88–1.56)	0.596	0.288	1.15(0.93–1.42)	0.389	0.201	**1.74(1.33–2.27)**	**0.346**	**0.000**
rs833061	3	618/673	1.09(0.93–1.28)	0.582	0.294	1.18(0.85–1.63)	0.809	0.325	1.01(0.79–1.29)	0.460	0.958	1.06(0.84–1.34)	0.484	0.620	**1.71(1.28–2.21)**	**0.794**	**0.000**
rs1570360 (-1154)	4	697/737	1.08(0.91–1.27)	0.541	0.390	1.23(0.85–1.76)	0.259	0.268	1.15(0.79–1.68)	0.170	0.453	1.05(0.85–1.30)	0.578	0.626	**1.92(1.36–2.72)**	**0.231**	**0.000**
rs833068	2	500/540	1.08(0.90–1.28)	0.909	0.402	1.28(0.85–1.94)	0.239	0.232	1.11(0.65–1.89)	0.071	0.713	1.05(0.82–1.35)	0.256	0.699	1.62(0.74–3.58)	0.061	0.230
rs3025020 (-583)	2	500/540	**1.24(1.03–1.50)**	**0.719**	**0.026**	**1.73(1.13–2.65)**	**0.920**	**0.011**	1.07(0.83–1.39)	0.823	0.604	1.18(0.93–1.51)	0.744	0.176	**2.65(1.77–3.97)**	**0.974**	**0.000**
rs3025039 (+936)	3	586/628	1.27(0.97–1.27)	0.276	0.087	0.87(0.35–2.18)	0.724	0.766	**1.43(1.05–1.96)**	**0.212**	**0.025**	**1.37(1.01–1.85)**	**0.235**	**0.042**	3.17(0.72–13.97)	0.004	0.128

*P*_h_: value of *Q*-test for heterogeneity test; *P*: *Z*-test for the statistical significance of the OR.

**Figure 5:**
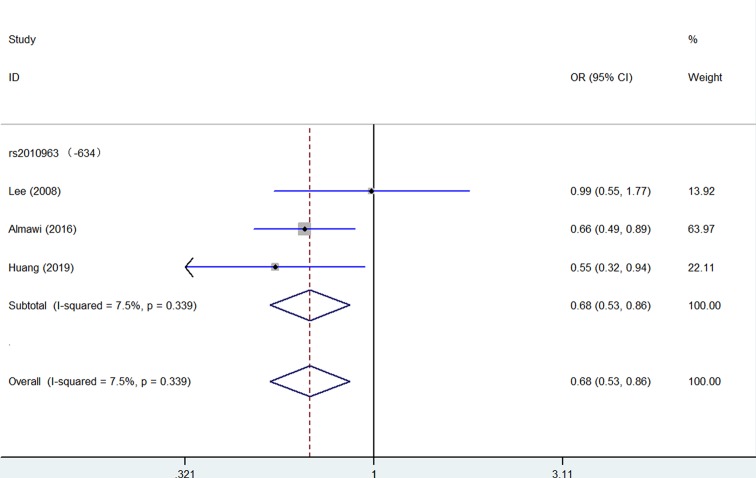
Forest plot of PCOS risk associated with *VEGF* gene polymorphism (rs2010963) (Heterozygote comparison) in the whole The squares and horizontal lines correspond to the study-specific OR and 95% CI. The area of the squares reflects the weight (inverse of the variance). The diamond represents the summary OR and 95% CI.

**Figure 6:**
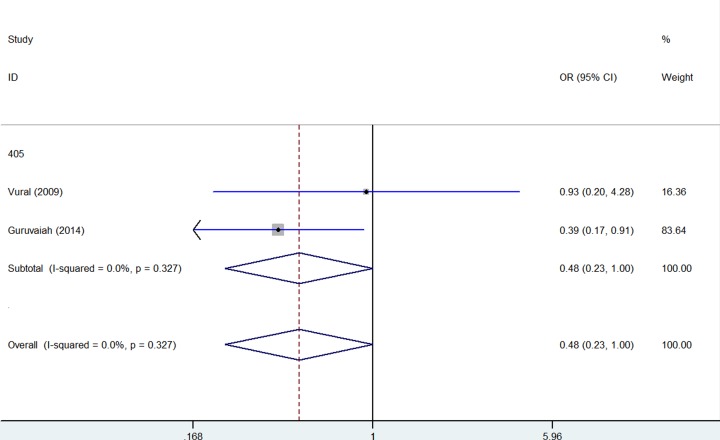
Forest plot of PCOS risk associated with *VEGF* gene polymorphism (+405) (Allelic contrast) in the whole The squares and horizontal lines correspond to the study-specific OR and 95% CI. The area of the squares reflects the weight (inverse of the variance). The diamond represents the summary OR and 95% CI.

In opposite, several *VEGF* gene SNPs acts as a decreased association or protective effect for PCOS risk: rs2010963 (Heterozygote comparison: OR = 0.68, 95% CI = 0.53–0.86, *P* = 0.339 for heterogeneity, *P* = 0.002, Figure 5, Table 2); +9812 (Allelic contrast: OR = 0.60, 95% CI = 0.43–0.83, *P* = 0.892 for heterogeneity, *P* = 0.002, Figure 3, Table 2); +405 (Homo*z*ygote comparison: OR = 0.48, 95% CI = 0.23–1.00, *P* = 0.327 for heterogeneity, *P* = 0.050, Figure 6, Table 2).

### Sensitivity analysis and bias diagnosis

We used a sensitivity analysis to determine whether modifying the meta-analysis inclusion criteria affected the results. No other single study influenced the summary OR qualitatively (data not shown). Egger and Begg’s tests were performed to assess publication bias and the funnel plot symmetry was examined. Finally, no publication bias was observed (data not shown).

### Gene–gene network diagram and interaction of online website

**Figure 7:**
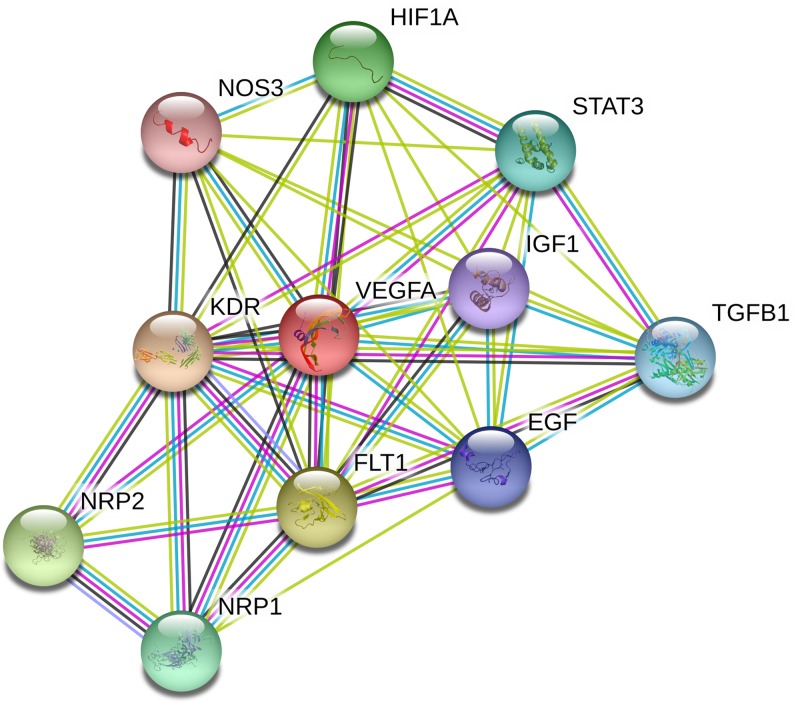
Human VEGF interactions network with other genes obtained from String server At least 10 genes have been indicated to correlate with VEGF gene. KDR: vascular endothelial growth factor receptor 2; FLT1: vascular endothelial growth factor receptor 1; NRP2: neuropilin-2; HIF1A: hypoxia-inducible factor 1-α; NRP1: neuropilin-1; STAT3: signal transducer and activator of transcription 3; TGFB1: transforming growth factor β-1; EGF: pro-epidermal growth factor; IGF1: insulin-like growth factor 1; NOS3: nitric oxide synthase, endothelial.

String online server indicated that VEGF gene interacts with numerous genes. The network of gene–gene interaction has been illustrated in Figure 7.

## Discussion

A strong association between increased serum VEGF levels and PCOS was previously reported, and a correlation between serum VEGF levels and increased ovarian stromal blood flow in women with polycystic ovaries was suggested [15,16,34,35]. On the other hand, polymorphisms in the VEGF gene may lead to alterations in the production of this protein and may play an important role in the pathophysiology of PCOS, contributing to ovulatory dysfunction, infertility, and ovarian hyperstimulation syndrome, which are commonly observed in women with PCOS [36].

To combine the important of genetic etiology of PCOS, it makes sense to deep study the *VEGF* gene polymorphisms. There are at least 80 SNPs places in this gene (NCBI Gene association no: NT 007592). Among them, we searched several popular databases to select more comprehensive case–control studies about SNPs in *VEGF* gene, which have been reported more than once about PCOS disease. Finally, 11 SNPs [rs2010963 (-634), +9812, +13553, -460, +405, rs699947 (-2578), rs833061, rs1570360 (-1154), rs833068, rs3025020 (-583), rs3025039 (+936)] were identified.

Polymorphisms in the promoter region (loci: -2578, -1154 and -460) or intron 6 (loci: -583) or 5′-untranslated region (loci: +405, +963 and -634) or +534 have been associated with different levels of VEGF expression. It was reported that -2578 C, -460 T and +405 G alleles appear to correlate with altered VEGF expression levels [18,20,37–40]. In addition, a strong association between increased serum VEGF levels and PCOS was previously reported, and a correlation between serum VEGF levels and increased ovarian stromal blood flow in women with polycystic ovaries was suggested [15,16,34,35]. Due to above items, these four SNPs have been widely reported in PCOS.

It is the first time to collect such more SNPs at one time, 11 SNPs containing 5203 cases and 5462 controls. The meaningful of our current analysis was that we found five SNPs (rs699947, rs833061, rs1570360, rs3025020, rs3025039) may act as a risk effect for the development of PCOS; moreover, three SNPs (rs2010963, +9812, +405) may have a protective influence for PCOS. Among above results, rs699947, rs3025020 and +405 polymorphisms were consistent with abnormal expression of VEGF gene in serum, and may be associated with PCOS risk through the serum *VEGF* levels. Some factors may be explained: First, different polymorphisms in the same gene may exert different effects on gene expression and function, leading to vary PCOS risks. Second, single genes or single environmental factors may not be likely to have direct effects on PCOS susceptibility, but complex interactions between genetic and environmental factors may be involved in the disease development. The last but not the least, if numbers of included studies were small, false-negative results may be detected for each polymorphism [41].

If one woman exists one or more significant following five SNPs (rs699947, rs833061, rs1570360, rs3025020, rs3025039) for VEGF from peripheral blood test, which may indicate that it is possible to increase the occurrence of PCOS for her in present time or at some point in the future. Therefore, it can be offer us some targets to intervene, such as lifestyle modification (reducing the BMI, obesity, high blood pressure, high blood fat and cardiovascular disease) for prevention status, regular gynecological examination (vaginal ultrasound or CT scans or endocrinology) to identify or rule out this disease and carry out treatments as soon as possible (oral contraceptive therapy, ovulation induction, high testosterone therapy, insulin sensitizer, GLP-I receptor agonist therapy, surgical treatment) [42]. To sum up, we wish to use this method to reduce the incidence of PCOS and improve the cure rate of early treatment. In addition, for another three decreased association SNPs (rs2010963, +9812, +405) and these no associated SNPs, it is not necessary to take corresponding monitoring measures at current moment.

In addition, we used the online analysis system-String to predict potential and functional partners (Figure 7). Finally, ten genes were predicted. The average score was very high. Among them, the highest score of association was KDR and FLT1 (score = 0.999); however, TGFB1, EGF, IGF1 and NOS3 had the lowest scores (0.993). The action of VEGF is mediated by binding to tyrosine kinase receptors, VEGFR-1 (Fms-like tyrosine kinase: FLT1) and VEGFR-2 (kinase domain-containing receptor: KDR) [43]. Pan et al. demonstrated that HIF1A-mediated VEGF expression might be an important mechanism regulating ovarian luteal development in mammals *in vivo*, which may provide new strategies for fertility control and for treating PCOS [44]. Additional, IGF-1, TGFB1, STAT3 and NOS3 were just suggested to participate in the development of PCOS [45–48], rather than combined with VEGF. Above information predicted FLT1, KDR, and HIF1A may influence VEGF and regulate the PCOS development, which maybe become intervention and treatment target genes in the future.

Several limitations in our current analysis should be considered. First, unadjusted OR was used. Second, control sources were not all health women. Third, only four databases were searched for study retrieval, few relevant studies may be omitted. Fourthly, gene–gene interaction was missing. Fifth, other confounding factors such as age, BMI, lifestyle, total cholesterol, free androgen index, triglycerides and environment were not included and analyzed.

In summary, in the present meta-analysis, *VEGF* gene polymorphisms may be associated with PCOS susceptibility.

## Abbreviations

CI: confidence intervals
OR: odds ratios
PCOS: polycystic ovary syndrome
SNP: single-nucleotide polymorphism
VEGF: vascular endothelial growth factor
